# Separation of saponins from quinoa bran by a novel single-column three-stage foam fractionation technology

**DOI:** 10.1016/j.fochx.2026.103954

**Published:** 2026-05-07

**Authors:** Lei Jia, Wei Liu, Xusheng Ge, Xiaoyan Miao, Kai Zhang

**Affiliations:** a College of Biochemistry and Environmental Engineering, Baoding University, Baoding 071000, Hebei, China; b Baoding Key Laboratory of Fungal Resource Development and Biomass Efficient Utilization, Baoding 071000, Hebei, China; c School of Chemical Engineering and Technology, Hebei University of Technology, No.8 Guangrong Road, Dingzi Gu, Hongqiao District, Tianjin 300130, China

**Keywords:** Foam fractionation, Quinoa saponins, Bubble layer, Separation efficiency

## Abstract

This study developed a novel single-column three-stage foam fractionation technology to separate saponins from quinoa bran. For efficient separating saponins from their highly concentrated aqueous solution (300 mg/L), large bubbles, multi-layered foam and small bubbles performed their own specific functions to achieve a high enrichment ratio of 7.1 and a high recovery percentage of 91.7%, respectively. Multi-layered foam could strengthen the foam drainage with a low liquid holdup of 0.5% and enhance the foam stability with a high half-life of 630 s simultaneously. FTIR and HPLC-MS were performed to analyze and characterize the ingredients of the product (mainly are triterpenoid saponins) and the purity of total saponins (91.9%). This work is expected to provide a simple and highly-efficient technology for the separation of natural surface-active substances and facilitate the industrial application of foam fractionation.

## 1 Introduction

Quinoa (*Chenopodium quinoa*, Willd.), as an annual grain native to South America, has been increasingly cultivated globally owing to its abundant nutritional value. The content of valuable ingredients (protein, fat, vitamins, saponins etc.) in quinoa seed is higher than those of most cereal crops (Qian et al., 2023; Yang, Guo, et al., 2024; Ng & Wang, 2021). Saponins are important secondary metabolites in quinoa and most of them have been identified in the outer layers of the quinoa seed (i.e. quinoa bran) (Tan et al., 2022). Given the diverse pharmacologic and biological activities, such as anti-tumor, anti-inflammatory, surface activity and hemolysis effect, saponins have attracted much attention from researchers (Dong et al., 2020; Kim et al., 2025; Shang et al., 2025). Previous researches have shown that the saponins in quinoa bran mainly belong to the triterpenoid glycosides or sapogenins (El Hazzam et al., 2020; Lim et al., 2020). However, the separation of saponins from quinoa bran has been rarely reported, and quinoa bran was mainly used in processing animal feed as by-products of quinoa processing (Yang, Liu, et al., 2024). Therefore, the efficient separation of saponins from quinoa bran is of great significance for the rational utilization of quinoa.

The separation process of saponins from plant parts consists of crude leaching and further purification. In the former process, the commonly utilized solvents include water, alcohol, methanol, and some deep eutectic solvents (Cai et al., 2023; Deng et al., 2025; Liu et al., 2025). Among them, water has become a more desirable solvent because of its simple operation and good environmental compatibility. Water will be hence selected as the leaching solvent in the current work. In the latter process, the techniques for separating saponins from the leaching liquor include column chromatography, ultrafiltration, macroporous resins adsorption and foam fractionation (Li et al., 2013; Wang et al., 2022; Wang et al., 2024). Among them, foam fractionation is a preferable technology because of its simple equipment, low cost and good environmental compatibility. Furthermore, the excellent surface activity of saponins endows them with superior foam property, a feature that lends them to the application of foam fractionation technology (Buckley et al., 2022). However, the separation efficiency of saponins will be unsatisfactory due to the excessively high concentration in leach liquor. The performance of foam fractionation is evaluated by enrichment ratio (*E*) and recovery percentage (*R*) (Liu et al., 2021). The saponins have good foamability and high concentration (several hundred milligrams per liter) in leaching liquor, resulting in low enrichment ratio (Jiang et al., 2016). However, if the leaching liquor is diluted before foam fractionation, the separation period will be prolonged due to the increased volume of the aqueous solution. Therefore, increasing the enrichment ratio was a great challenge for separating highly concentrated surfactant by foam fractionation. Previous researchers have developed many methods to address the problem, such as increasing temperature, adopting two stage foam fractionation technology and coupling with adsorption (Chang et al., 2023; Hu et al., 2019; Yan et al., 2011). However, the applications of above methods were limited by the high cost and complex equipment. Therefore, novel foam fractionation technologies are urgently required to be developed for separating the high-concentration saponins from leaching liquor.

In foam fractionation, bubble size is a key parameter because it affects two essential processes that determine the performance of foam fractionation: interfacial adsorption and foam drainage. At a given volumetric air flow rate, the larger bubbles lead to a greater drainage rate, while the smaller bubbles enhance the available surface area for the adsorption of surfactant molecules (Alam et al., 2024; Narsimhan & Ruckenstein, 1986). In our previous work, for efficient separation of carbon nanotubes, mono-layered foam in a short foam phase was created by placing large-bubble layer below small-bubble layer (Jia et al., 2022). Ultimately, the foam drainage was strengthened due to the weakened capillary phenomenon inside small-bubble layer. By the same principle, multi-layered foam (MLF) would be created and studied in a tall foam phase in this work. Then, based on the bubble size and MLF, a novel foam fractionation technology where large bubbles, MLF and small bubbles perform their own specific functions would be developed to separate saponins. In this technology, only one column was used and foam fractionation process was divided into three consecutive stages to address the highly concentrated saponins aqueous solution.

In this work, a novel single-column three-stage foam fractionation technology (STFFT) was developed to improve the separation efficiency of saponins from leaching liquor. The optimal extraction conditions were firstly determined. Then, foam fractionation technology has been systematically studied, including foam fractionation conditions, MFL and STFFT. Finally, the product was analyzed and characterized by Fourier transform infrared spectrometer (FTIR) and liquid chromatography-mass spectrometer (HPLC-MS). This study aimed at providing an efficient process technology with simple equipment and low costs for the separation of saponins.

## 2 Materials and methods

### 2.1 Materials and reagents

The quinoa bran powder was supported by Qinghai Dongtu Xiyu Network Technology Co., Ltd., China. Vanillin was purchased from Shanghai Maclean Biochemical Technology Co., Ltd., China. Perchloric acid and glacial acetic acid were purchased from Tianjin Damao Chemical Reagent Factory, China. All the above reagents were analytical reagents.

### 2.2 Extraction and initial purification of saponins from quinoa bran

Quinoa bran powder was added into deionized water and then magnetic stirring for leaching saponins under the preset experimental conditions. The purity of saponins inside the crude leaching liquor was at a low level (20 % approximately) (Zhou et al., 2023). Absolute ethyl alcohol was added into the leaching liquor to precipitate polysaccharide, protein, inorganic salt etc. Finally, the initially purified leaching liquor was obtained (the purity of saponins more than 40 %) (Bustos et al., 2024).

### 2.3 Measurement of the saponins concentration

The vanillin-perchloric acid colorimetry was used to detect the concentration of saponins. The maximum absorption wavelength was 560 nm determined by 752 N-UV spectrophotometer (Chan & Liang, 2025). The standard equation was *A* = 0.90657*C* + 0.0118, *R*^2^ = 0.99969, where *A* is the absorbance, *C* is the concentration of saponins ranging from 0 g/L to 0.04 g/L. *R*^2^ is the linear correlation index.

### 2.4 Separation of saponins from the leaching liquor by foam fractionation

#### 2.4.1 Foam fractionation experiments

**Fig. 1:**
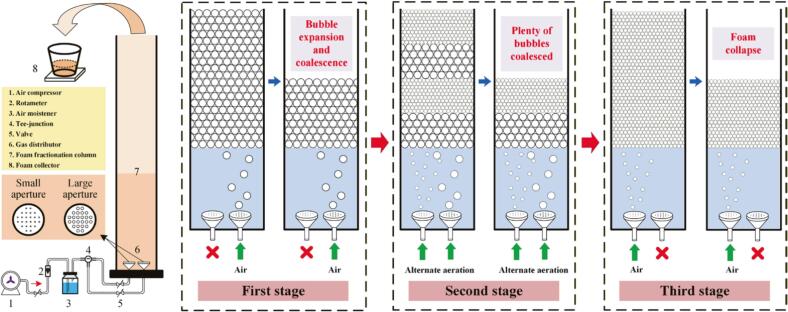
Schematic diagrams of foam fractionation equipment and STFFT.

As shown in Fig. 1, a transparent acrylic tube with 500 mm in height and 40 mm in inner diameter was served as foam fractionation column. The initial loading liquid height was 300 mm. Three gas distributors with different average pore diameters (0.425 mm, 0.180 mm and 0.125 mm) were used in this work and two of them were installed at the bottom of the column in one foam fractionation. The average size of bubbles generated by three gas distributors were determined by the method of Hofmann et al. (2015). The results are 1.03 mm, 0.66 mm and 0.31 mm, respectively. Airflow was generated by an air compressor and air was introduced into column through one of the two gas distributors after moistened. A rotameter and a tee-junction were applied to control air flow rate and air flow direction respectively. With the air continuously pumped into column, the foam was collected from the top of column and then the foamate could be obtained after foam collapse.

MLF was created by introducing small bubbles and large bubbles into the column alternatively. Air was firstly pumped into the column through small aperture gas distributor to form small-bubble layer in the foam phase. Then, controlling air flows through large aperture gas distributor to form large-bubble layer just beneath the small-bubble layer. Repeating the above two processes and the MLF was created.

STFFT was designed to simplify equipment and separation process. In first stage, air was pumped into the column through large aperture gas distributor to generate large bubbles, which were applied to cope with the high concentration of saponins. When the bubble expansion or bubble coalescence occurred, the first stage was terminated. In second stage, MLF was applied to cope with the low drainage rate of small-bubble layer and the unstable large-bubble layer. With the concentration of saponins continuously decreased, plenty of bubbles began to coalesce, causing the MLF difficult to remain stable. In third stage, small bubbles were applied in the separation process until the foam no longer overflowed from the top of the column. The third stage has come to an end and the foam fractionation process has been completed.

#### 2.4.2 Measurement of liquid holdup and half-life period

Liquid holdup (*ε*) (Liu et al., 2013) is the water content of foam and it could determine the enrichment ratio of saponins. MLF was used for strengthening foam drainage, expecting to decrease the liquid holdup of small-bubble layer and meanwhile to enhance the foam stability of large-bubble layer. Therefore, liquid holdup was calculated by Eq. (1). Foam stability was evaluated by half-life period of foam (*t*_1/2_) (Moreno et al., 2021), which is defined as the elapsed time that the foam's height declined to half of its initial height.

(1) $$\varepsilon =\frac{V_{f}}{V_{f}+V_{g}}$$

where *V*_*f*_ is the foamate volume (obtained after the collected foam collapsed), (L). *V*_g_ is the air volume introduced into column, (L).

#### 2.4.3 Evaluation of separation efficiency of STFFT

The separation efficiency of STFFT for highly concentrated saponins was evaluated by enrichment ratio (*E*) and recovery percentage (*R*), which are defined as Eq. (2) and Eq. (3).

(2) $$E=\frac{C_{f}}{C_{0}}$$

(3) $$R=\frac{C_{f}V_{f}}{C_{0}V_{0}}$$

where *C*_0_ is the initial concentration of saponins, (mg/L). *V*_0_ is the initial volume of saponins solution, (L). C_f_ is the concentration of foamate, (mg/L). *V*_*f*_ is the foamate volume, (L).

### 2.5 Analysis and characterization of saponins

FTIR and HPLC-MS were used to analyze and characterize the ingredients of product separated by foam fractionation (Chae et al., 2025). FTIR spectra were measured using a KBr matrix and the scanning range from 4000 cm^−1^ to 400 cm^−1^. HPLC was measured on a Symmetry™ C_18_ column (4.6 × 100 mm,3.5 μm, Waters Sunfire) with mobile phase A: formic acid/deionized water, *v*/v = 1:100 and mobile phase B: formic acid/acetonitrile, v/v = 1:100 (0–7 min, 95%A + 5%B; 7–12 min, 5%A + 95%B; 12–15 min, 95%A + 5%B) at flow rate 0.8 mL/min and 30 °C. A UV-detector was applied to measure saponins at 214 nm. Then, MS analysis would be obtained by ionizing each ingredient in the electrospray ionization and operating in positive mode.

## 3 Results and discussion

### 3.1 Extraction of saponins from quinoa bran

**Fig. 2:**
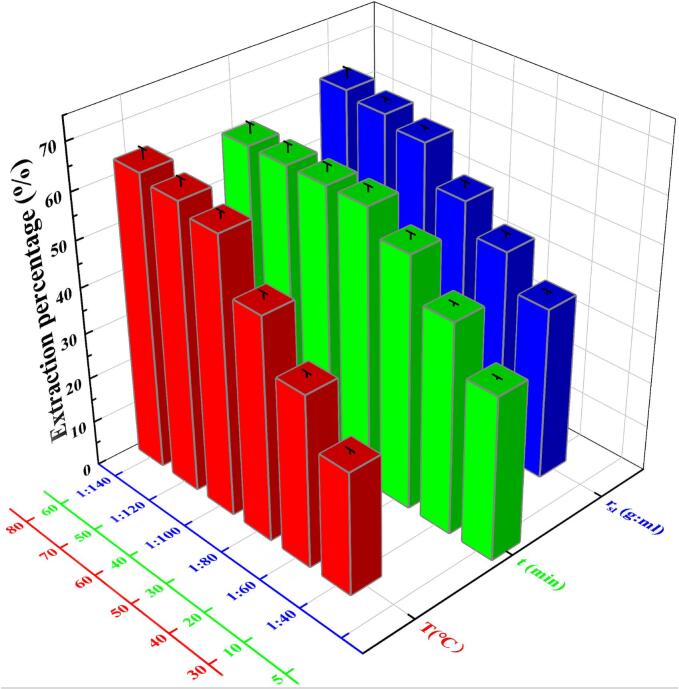
Effects of temperature (T), extraction time (t), and ratio of solid-to-liquid (r_sl_) on extraction percentage of saponins.

In order to extract saponins as many as possible, three important extraction factors were optimized. As shown in Fig. 2, the extraction percentage of saponins increased with the increasing of temperature (the red section). High temperature could accelerate the diffusion of saponin molecules into aqueous phase, thereby facilitating the extraction of saponins (reached to 64.0%). As seen in green section of Fig. 2, prolonging the extraction time resulted in an increased extraction percentage (reached to 60.6%) until the diffusion of saponin molecules from quinoa bran to the aqueous phase reached equilibrium. In addition, an appropriate solid-to-liquid ratio (the blue section) is essential for achieving the maximum saponins yield (reached to 64.0%) because a given volume of the aqueous phase could only solubilize a finite amount of saponins. Considering profit maximization, the optimal extraction conditions of saponins were determined. It could draw a conclusion from Fig. 2 that the optimal extraction conditions were temperature 60 °C, extraction time 30 mins and solid-to-liquid ratio 1:100 (g:ml). The maximum extraction percentage of saponins from quinoa bran reached 60.3% and the saponins concentration in leaching liquor was 700 mg/L approximately. This result demonstrated that the saponins contained in quinoa bran have a high content and are easy to obtain.

### 3.2 Foam fractionation

The excessively high concentration and excessive foaming capacity of saponins could result in low separation efficiency due to the increased volume of entrained liquid during the foam fractionation process. However, diluting the leaching liquor increases its volume severalfold, thereby posing significant inconvenience to subsequent saponins separation. To overcome the above challenges, STFFT was adopted to efficiently separate saponins from the leaching liquor.

#### 3.2.1 Optimization of foam fractionation conditions

**Fig. 3:**
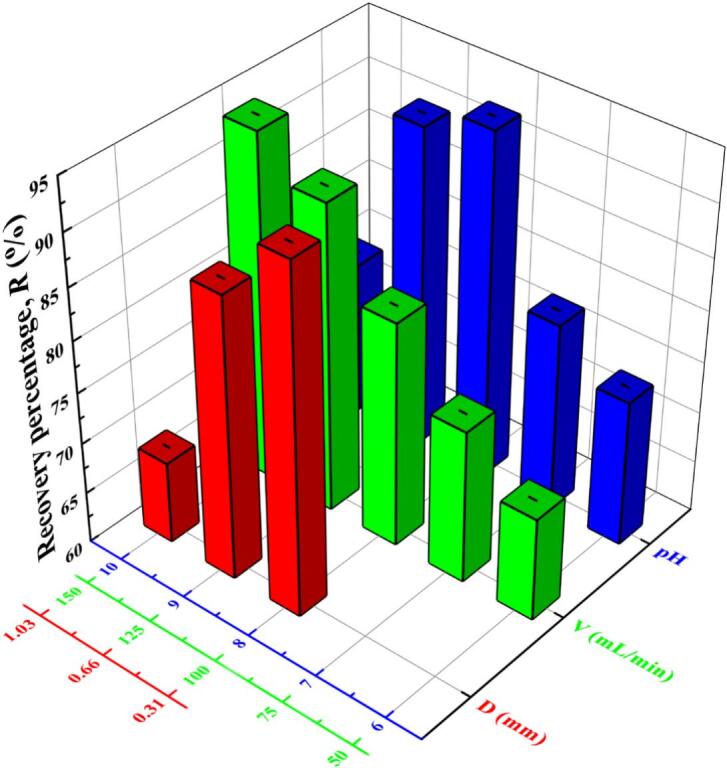
Effects of bubble diameter (*D*), volumetric air flow rate (*V*), and pH on recovery percentage of saponins by foam fractionation.

**Fig. 4:**
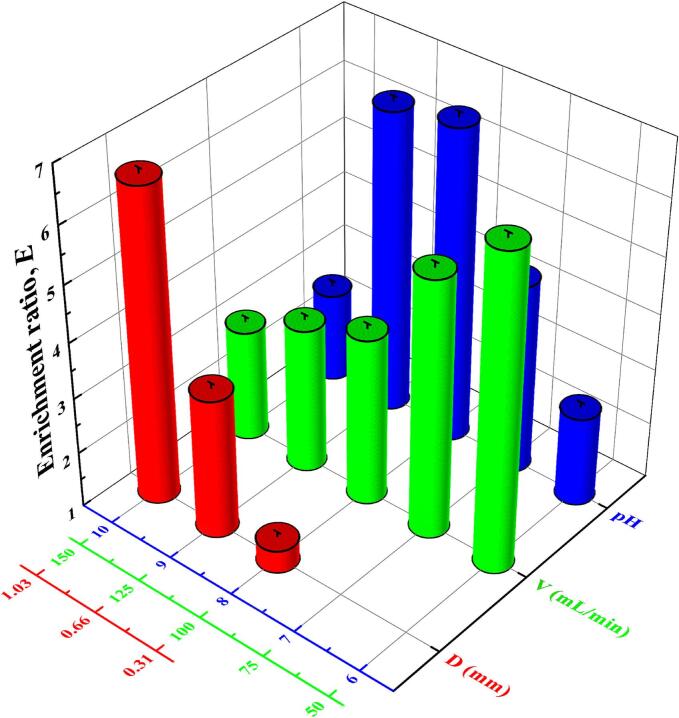
Effects of bubble diameter (D), volumetric air flow rate (V), and pH on enrichment ratio of saponins by foam fractionation.

The effects of average bubble diameter (*D*, the red section), volumetric air flow rate (*V*, the green section) and pH (the blue section) on recovery percentage and enrichment ratio were investigated under the conditions of saponins concentration 300 mg/L. The results in Fig. 3 and Fig. 4 indicated that large bubbles were contributed to enhance the enrichment ratio (6.6) while small bubbles were contributed to enhance the recovery percentage (93.7%). Above results could be explained that the drainage of foam constituted by large bubbles was strengthened by wide plateau borders inside foam phase (Jia et al., 2021). Whereas small bubbles had high specific surface area which could accommodate more saponins molecules (Zhang et al., 2021). Increasing volumetric air flow rate could accelerate the rising speed of the foam and then the large volume of liquid contained saponins was collected from the top of foam fractionation column. Therefore, the saponins contained in entrained liquid made the recovery percentage increase (reached to 93.6%) while the large volume liquid made the enrichment ratio decrease (dropped to 3.0). For achieving 80% recovery percentage of saponins, 125 mL/min was chosen as the suitable volumetric air flow rate. The variation trend of the recovery percentage was similar to that of the enrichment ratio when the pH changed from 6 to 10. The functional groups in saponin molecules could be protonated in a highly acidic environment, thus weakening the foaming performance of saponins. In strong alkaline environment, the structure of the saponin molecule could be destroyed by hydrolysis (Böttcher & Drusch, 2016; Savarino et al., 2022). Acidic or strong alkaline environment decreased the recovery percentage (dropped to 74.3%) and enrichment ratio (dropped to 2.6) of saponins simultaneously. Therefore, the optimal pH for the separation of saponins was 8.

#### 3.2.2 MLF for strengthening foam drainage and enhancing foam stability

**Fig. 5:**
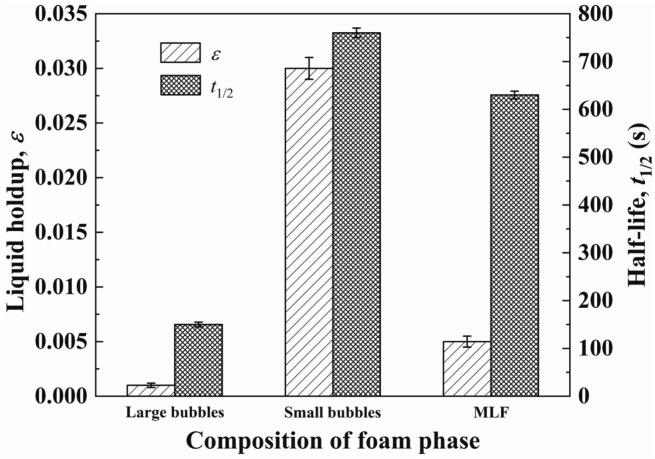
Effects of Large bubbles, Small bubbles and MLF on liquid holdup and half-life of foam.

**Fig. 6:**
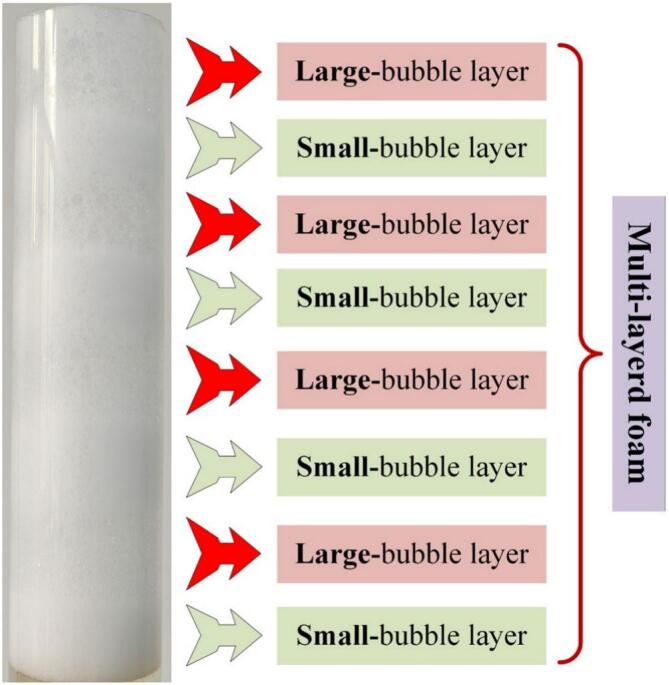
Photograph of multi-layered foam.

Large bubbles in the foam phase have a high foam drainage rate and are prone to rupture because of coalescence. In contrast, small bubbles exhibit a high interfacial adsorption capacity at the gas-liquid interface but a low foam drainage rate, attributed to the narrow plateau borders between adjacent bubbles (Rüegg et al., 2022). To overcome above problems, MLF was created for strengthening foam drainage of small-bubble layer and enhancing foam stability of large-bubble layer. The average bubble size of 1.03 mm (large bubbles) and 0.31 mm (small bubbles) were adopted in this section to create the MLF under the conditions of volumetric air flow rate 125 mL/min, saponins concentration 300 mg/L and pH 8. The results and the photograph of MLF are presented in Fig. 5 and Fig. 6 respectively.

Fig. 5 showed that the liquid holdup of foam composed of large bubbles (0.1%) was significantly lower than that of foam composed of small bubbles (3.0%). However, the stability of large bubbles (*t*_1/2_ = 150 s) was much poorer than that of small bubbles (*t*_1/2_ = 760 s). Based on the above results, the large bubbles and the small bubbles were alternately introduced to create MLF. Above the liquid phase surface, large-bubble layer placed beneath small-bubble layer could weaken capillary phenomenon inside the small-bubble layer, thus strengthening foam drainage (Jia et al., 2021). The liquid holdup of foam was 0.5%, which was 83.3% lower than that of small bubbles. Meanwhile, the entrained liquid flowing from the small-bubble layer into the large-bubble layer effectively maintained the stability of large bubbles by supplementing liquid in the plateau borders. With the foam rising in the column, a new small-bubble layer was generated beneath the large-bubble layer, which could weaken the foam drainage of the large-bubble layer. A relatively stable level of liquid holdup reduced the coalescence and breakage of large bubbles, thereby maintaining the foam stability of large-bubble layer. The half-life of foam was 630 s, which was 320% higher than that of large bubbles. The alternating arrangement of small-bubble layers and large-bubble layers resulted in the foam being both dry and stable. Fig. 6 showed that the light transmittance of small-bubble layer was lower than that of large-bubble layer. Small-bubble layer possesses high liquid holdup and complex structure which could make it absorb light, refract light and reflect light. It could be seen from Fig. 6 that small amounts of bubbles were still undergoing expansion or coalescence as they rose in the column. This result could be explained by the low liquid holdup of foam, indicating that foam drainage was strengthened by MLF. Other foam fractionation processes that require strengthened foam drainage can also adopt MLF.

#### 3.2.3 Design of STFFT

**Fig. 7:**
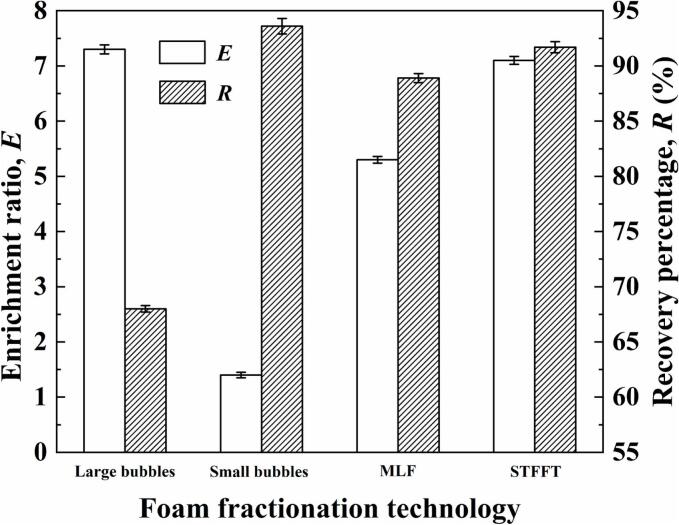
Effects of foam fractionation technology on the enrichment ratio and recovery percentage of saponins.

**Fig. 8:**
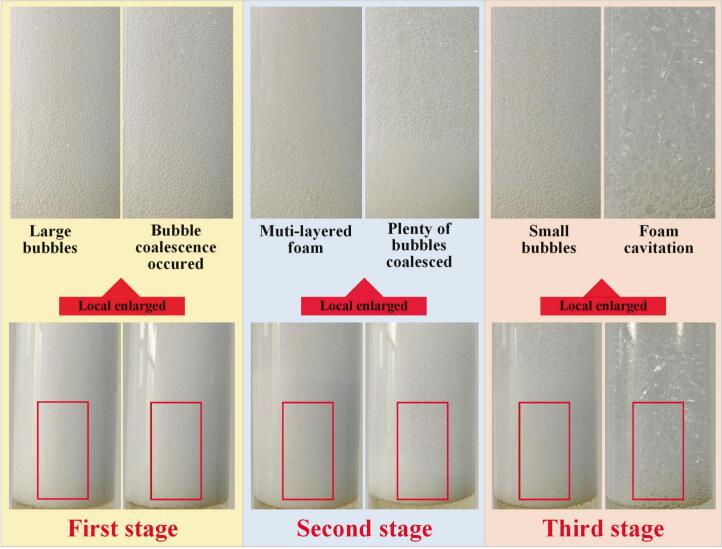
Photographs of the start and end moments of the three stages.

It could be detected from section 3.2.1 that achieving high enrichment ratio and high recovery percentage of saponins simultaneously was difficult in foam fractionation. To deal with this problem, STFFT was designed based on the advantages of large bubbles, MLF and small bubbles. The results are presented in Fig. 7. Fig. 8 showed the foam photographs of the three stages.

It could be seen from Fig. 7 that both MLF and STFFT could increase the enrichment ratio (5.3 and 7.1) and the recovery percentage (88.9% and 91.7%) of saponins simultaneously. Moreover, STFFT demonstrated a more remarkable improvement on the separation efficiency of saponins. At the beginning of the foam fractionation process, high concentration of saponins made the bubble extremely stable due to the high surface excess of saponins. Evidently, the extremely stable foam with high water-holding capacity was not conducive to achieving a high enrichment ratio. Therefore, in the first stage of STFFT (seen in Fig. 8), large bubbles were applied in foam fractionation to preliminarily decrease the concentration of saponins. With the continuous reduction of saponins concentration, bubble expansion and coalescence occurred because the large bubbles became unstable. At this juncture, the emergence of larger bubble marked the end of the first stage. In second stage, the concentration of saponins was still in a relatively high level for small bubbles. MLF was adopted to strengthen the foam stability of large-bubble layer and foam drainage of small-bubble layer (seen in section 3.2.2). When the MLF was unable to maintain stability (i.e. plenty of bubbles coalesced), the second stage was terminated. In third stage, the concentration of saponins was at a low level. Small bubbles were applied to enhance the recovery percentage of saponins. The stability of small bubbles was higher than that of large bubbles because small bubbles possess higher surface excess than those of large bubbles under the same conditions. Therefore, using small bubbles could separate more saponins from feeding solution. Notably, bubble coalescence persisted throughout the entire third stage, leading to the rare observation of the small bubbles. When the foam collapsed and ceased to overflow from the top of the column, the third stage has terminated and the foam fractionation process has been completed. In conclusion, large bubbles were used to preliminarily decrease the concentration of saponins; MLF was used during the transitory stage; small bubbles were used to separate saponins as many as possible. The final enrichment ratio and recovery percentage of saponins reached 7.1 and 91.7% respectively. The natural substances with strong foaming properties can be extracted and recycled through this technology.

### 3.3 Characterization and analysis of the product

**Fig. 9:**
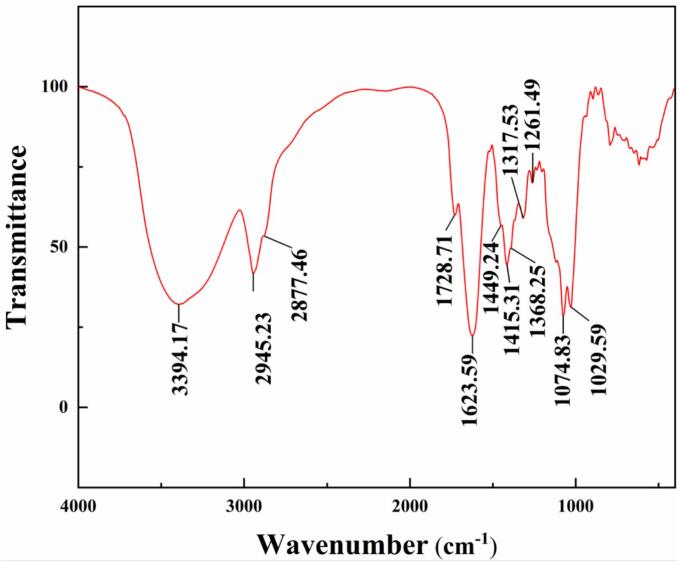
FTIR spectra of the foam fractionation product.

**Fig. 10:**
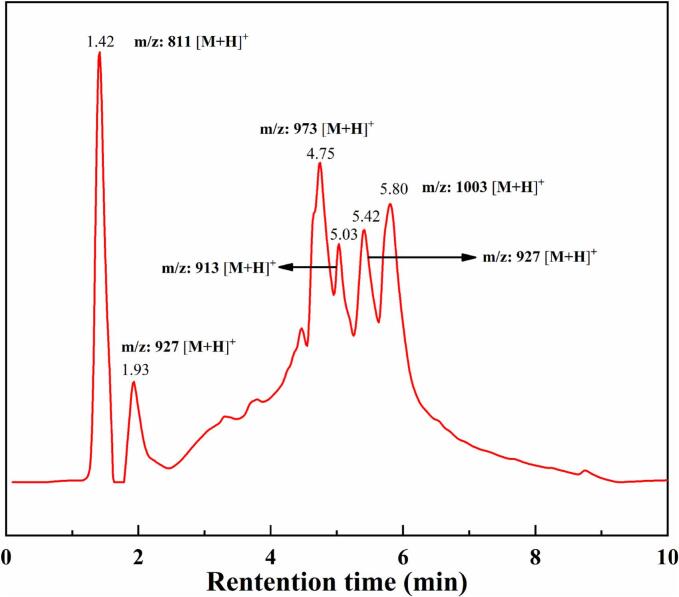
ESI-total ion chromatogram of the foam fractionation product.

The main ingredients of saponins from quinoa bran are triterpene saponins (Nie et al., 2009). The product was analyzed by Fourier transform infrared spectrometer (FTIR) and liquid chromatography-mass spectrometer (HPLC-MS). The results are presented in Fig. 9 and Fig. 10.

It was found in Fig. 9 that the product had OH (∼3394.17 cm^−1^), CH_2_ (∼2877.46 cm^−1^, ∼2945.23 cm^−1^), CH_3_ (∼1368.25 cm^−1^, ∼1449.24 cm^−1^), C=O (∼1728.71 cm^−1^), C-O-C and carboxylic ester (∼1029.59 cm^−1^, ∼1074.83 cm^−1^, ∼1261.49 cm^−1^), C=C (∼1415.31 cm^−1^, ∼1623.59 cm^−1^) groups. The absorption peaks at ∼2877.46 cm^−1^ and ∼ 2945.23 cm^−1^ stronger than that at ∼1368.25 cm^−1^ and ∼ 1449.24 cm^−1^ indicated more CH_2_ groups than CH_3_ groups in the product. The strong absorption peaks of the above groups could be attributed to abundant glycosides in the product (Yang et al., 2018). Compared with the FTIR spectrum of oleanolic acid reported in the literature, the spectral curve of the product exhibited significant similarity, which confirmed that the product was sapogenin (i.e. saponins) (Almutairi & Ali, 2015). In Fig. 10, It was found that the molecular formulas of triterpenoid saponins in the product concluded C_42_H_66_O_15_ (*m*/*z*: 811 [M + H]^+^), C_47_H_74_O_18_ (m/z: 927 [M + H]^+^), C_48_H_76_O_20_ (m/z: 973 [M + H]^+^), C_47_H_76_O_17_ (m/z: 913 [M + H]^+^), C_47_H_74_O_18_ (m/z: 927 [M + H]^+^), C_49_H_78_O_21_ (m/z: 1003 [M + H]^+^). It was reported that the main aglycones in triterpene saponins of quinoa bran are Oleanolic acid, Hederagenin, Phytolaccagenicacid and Serjanicacid etc. (Lim et al., 2020). Oleanolic acid and Gypsogenin have isomerides and their molecular formulas are C_47_H_74_O_18_. The triterpenoid saponins contained in the product were consistent with the results of previous research. The HPLC analysis indicated that the peak area percentage of the saponins in the product was as high as 91.9%. Only a small number of unknown components have been hypothesized to be other types of saponins or other surface-active materials. Therefore, the saponins were effectively separated and purified from quinoa bran by the STFFT.

## 4 Conclusion

In this work, saponins were efficiently separated by the single-column three-stage foam fractionation technology. The quinoa bran contains a high content of saponins and water can be an excellent extractant. MLF could strengthen the foam drainage of small-bubble layer and enhance the foam stability of large-bubble layer simultaneously by utilizing the respective characteristics of large-bubble layer and small-bubble layer. Compared with other processes, enrichment ratio and recovery percentage could be increased simultaneously by STFFT. Large bubbles, MLF and small bubbles perform their own specific functions that correspond to separating high concentration of saponins, medium concentration of saponins and low concentration of saponins. The three stages were implemented in a single separation column, which not only simplifies the equipment but also reduces energy consumption. Applying this technology, an enrichment ratio of 7.1 and a recovery percentage of 91.7% with a high purity of 91.9% were obtained. This work provides a valuable reference for the separation of natural surface-active substances and the industrial application of foam fractionation. In future studies, multi-stage technologies can be developed to deal with complex systems.

## CRediT authorship contribution statement

**Lei Jia:** Writing – original draft, Investigation. **Wei Liu:** Writing – review & editing. **Xusheng Ge:** Investigation. **Xiaoyan Miao:** Project administration. **Kai Zhang:** Investigation.

## Declaration of competing interest

The authors declare that they have no known competing financial interests or personal relationships that could have appeared to influence the work reported in this paper.

## Data Availability

Data will be made available on request.
